# Health management gaps and EHR willingness among ethnic minority residents with rural backgrounds in Yunnan, China: a mixed-methods study

**DOI:** 10.3389/fpubh.2026.1923068

**Published:** 2026-08-26

**Authors:** Zetian Wang, Lingling Zheng

**Affiliations:** Faculty of Physical Education, Yunnan Minzu University, Kunming, Yunnan, China

**Keywords:** digital health, electronic health records, ethnic minority residents, public health services, rural communities, Yunnan Province

## Abstract

**Background:**

Digital health monitoring and electronic health records (EHRs) are increasingly used to support chronic disease management and community-based public health services. However, their implementation in rural ethnic minority communities depends not only on technical availability, but also on residents’ service contact, perceived usefulness, digital support, and expectations of feedback.

**Objective:**

This study examined health management gaps, digital health exposure, and EHR willingness among ethnic minority residents with rural community backgrounds in Yunnan Province, Southwest China. Methods: An explanatory sequential mixed-methods design was used. The quantitative phase comprised a community-based cross-sectional survey using a community-based non-probability sampling strategy combining on-site convenience recruitment with participant referral (snowball sampling). After data screening, 567 valid questionnaires were retained. Descriptive statistics and hierarchical binary logistic regression were used to examine factors associated with EHR willingness. The qualitative phase comprised semi-structured interviews with 21 residents to explain selected quantitative findings.

**Results:**

Routine health management gaps were evident. Overall, 60.3% of participants reported no annual health check-up, 72.3% reported no or unclear exposure to free health check-ups in the past year, and 45.7% were unwilling to establish an EHR. In the final regression model, EHR willingness was associated with free health check-up experience in the past year (aOR = 2.14, 95% CI: 1.33–3.46), perceived importance of chronic disease management (aOR = 2.41, 95% CI: 1.64–3.54), and awareness of community-based internet-enabled chronic disease management services (aOR = 1.72, 95% CI: 1.16–2.55). Interviews suggested that EHR willingness was shaped by offline service continuity, practical barriers to using digital tools, and expectations of useful feedback after health information collection.

**Conclusion:**

Digital health monitoring in rural ethnic minority communities should not be introduced as a stand-alone technical system. EHR implementation may be more acceptable when integrated with routine public health services, community-level digital support, and clear feedback mechanisms.

## 1 Introduction

Digital health has become an important part of contemporary public health practice, particularly in health information recording, chronic disease follow-up, and community-based health management (1). However, its value depends less on the presence of a technical platform than on whether it can be connected with routine services and residents’ everyday health practices (2). The World Health Organization has cautioned that digital health interventions should not be viewed as substitutes for functioning health systems, but as tools that need to be embedded within service delivery, resource allocation, acceptability, feasibility, and equity considerations (3).

This issue is especially relevant in rural ethnic minority communities, where health management often involves more than individual disease status (4). Previous work on comprehensive health among ethnic minorities has emphasized that health-related needs are shaped by behavioral habits, psychological and social adaptation, physical status, basic public health conditions, and living environments. It also points to the importance of health monitoring, health education, and service accessibility in ethnic minority areas (5, 6). Instead of treating these dimensions as a new theoretical model in the present study, they are used here as a background for understanding why routine health management and digital health readiness may vary across rural ethnic minority residents.

Yunnan Province provides an important empirical setting for examining these issues. According to the Seventh National Population Census of Yunnan Province, ethnic minority residents accounted for 33.12% of the provincial population, and the rural population accounted for 49.95% of the provincial population (7). These demographic features make Yunnan a relevant context for studying how health services and digital health tools reach ethnic minority residents with rural community backgrounds. At the same time, rural residence, mobility for work or study, and uneven access to community-level services may create differences in residents’ contact with routine health management and digital health support (8).

Electronic health records are often regarded as a foundation for continuous health monitoring because they can connect health information collection with follow-up, service coordination, and chronic disease management (9). Yet EHR willingness is not only a technical matter. Studies on health information technology acceptance suggest that perceived usefulness, ease of use, trust, and privacy concerns influence whether people accept electronic health systems. Research on electronic health data also shows that public trust in how health systems protect and use data is closely related to willingness to allow such data to be used (10, 11). For rural ethnic minority residents, these issues may be further complicated by limited exposure to digital tools, uneven service contact, and uncertainty about whether health data collection will lead to feedback or practical benefits (12).

Existing discussions on health monitoring for ethnic minority populations have paid attention to the types of data that may be collected, including demographic information, health behaviors, physical status, public health conditions, chronic disease awareness, and living environments (13). These categories are useful for organizing health information, but they do not by themselves explain whether residents are ready to participate in digital health monitoring. Readiness requires not only information systems, but also routine service contact, perceived usefulness, digital tool support, and confidence that health information will be returned to residents through meaningful feedback or follow-up (14). This distinction is important in rural ethnic minority communities, where digital health exposure may depend on community services, family support, or practical ability to use smartphones and apps (15).

Despite growing interest in digital health monitoring, limited evidence is available on EHR willingness among ethnic minority residents with rural community backgrounds. In particular, it remains unclear whether public health service contact, perceptions of chronic disease management, and community-level digital health exposure are associated with EHR willingness, and how residents themselves interpret these relationships. To address this gap, the present study had three objectives:

1 To describe health management gaps and digital health readiness among ethnic minority residents with rural community backgrounds in Yunnan Province;
2 To examine factors associated with EHR willingness, with particular attention to public health service contact, perceptions of chronic disease management, and community digital health exposure;
3 To use resident interviews to explain selected quantitative findings and identify practical barriers to integrating digital health monitoring into public health services for rural ethnic minority communities.

This study is also relevant to Sustainable Development Goal target 3.8, which emphasizes universal health coverage and access to quality essential health-care services. Examining health management gaps and EHR willingness among underserved rural ethnic minority communities can help identify practical barriers to equitable and continuous health-service access (16).

## 2 Methods

### 2.1 Study design and setting

This study adopted an explanatory sequential mixed-methods design, consisting of a cross-sectional quantitative survey followed by an explanatory qualitative phase. The study was conducted among ethnic minority residents with rural community backgrounds in Yunnan Province, Southwest China. The quantitative phase examined routine health management gaps, digital health exposure, and EHR willingness.

The qualitative phase was conducted after preliminary analysis of the survey data. Semi-structured interviews were used to explain selected quantitative findings, particularly the associations of free health check-up experience, perceived importance of chronic disease management, and community-based internet-enabled chronic disease management services with EHR willingness. The sequential design allowed the study to move from identifying statistical patterns to interpreting the service experiences, practical barriers, and expectations underlying those patterns.

**Figure 1:**
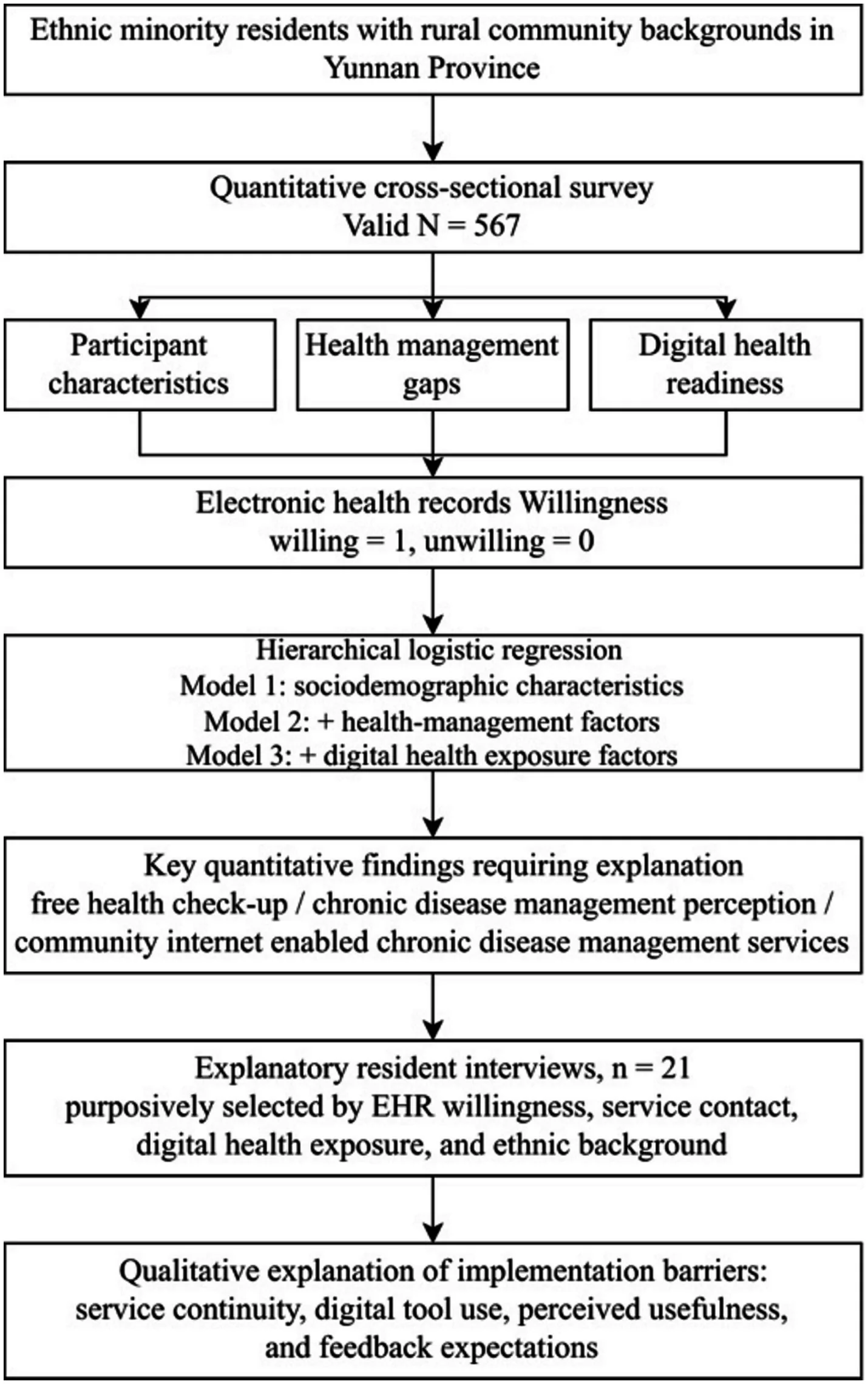
Workflow of the study.

Quantitative reporting was guided by the STROBE statement, and the integration of the quantitative and qualitative phases followed an explanatory sequential mixed-methods logic (Figure 1).

### 2.2 Participants and sampling

Participants were ethnic minority residents aged 17 years or above with rural community backgrounds in Yunnan Province. Residents were eligible if they self-identified as belonging to an ethnic minority group, had a household, family residence, or primary community background linked to rural ethnic minority communities in Yunnan, and were able to complete the questionnaire independently or with non-leading assistance from the research staff. Some participants were temporarily living, working, or studying in urban areas at the time of the survey, but were included if their family residence or primary community background remained connected to rural ethnic minority communities.

A community-based non-probability sampling strategy combining on-site convenience recruitment with participant referral (snowball sampling) was used. Initial participants were approached through on-site convenience recruitment and were then invited to forward the questionnaire link or QR code to eligible family members or other residents with the same rural ethnic minority community background. Initial recruitment was concentrated in three areas of Yunnan Province: Kunming in central Yunnan, Dehong Prefecture in western Yunnan, and Honghe Prefecture in southeastern Yunnan. Eastern Yunnan was not included because the sampling plan focused on areas with more concentrated ethnic minority communities. This approach helped reach eligible residents who were not present at the initial recruitment sites, including family members temporarily working, studying, or living outside their home communities. Because the questionnaire could circulate through participants’ family and social networks, the exact number recruited from each area, the number of referral waves, and the final balance between on-site and referred respondents could not be determined.

Before completing the questionnaire, participants were informed of the study purpose, voluntary participation, anonymity, and the right to withdraw. No personal identifiers such as names, identity card numbers, or telephone numbers were collected. Because the questionnaire link could be forwarded through snowball recruitment, the total number of eligible individuals who received the invitation was unknown and a conventional response rate could not be calculated. A total of 620 questionnaire submissions were recorded. Fifty incomplete submissions were removed during the initial screening, leaving 570 completed questionnaires. Three additional records were excluded because they were completed in less than 30 s or contained invalid or unclear information on key demographic variables such as age or gender. The final analytical sample therefore consisted of 567 valid questionnaires. Of all submitted questionnaires, 91.9% were complete and 91.5% were retained for analysis; these proportions should not be interpreted as population response rates.

### 2.3 Survey instrument and variables

A researcher-developed structured questionnaire was used to collect information on sociodemographic characteristics, health behaviors, routine health management, public health service contact, digital health exposure, chronic disease management perceptions, and EHR willingness. The questionnaire was designed to describe individual indicators rather than measure latent psychological constructs. Accordingly, items were analyzed separately and were not combined into composite scale scores. No existing validated instrument was adopted or adapted for this purpose.

The questionnaire was reviewed by three experts with backgrounds in ethnology, demography, and public health. They assessed the relevance of the items to the study objectives, the clarity of the wording and response options, and their contextual appropriateness for ethnic minority residents with rural community backgrounds. Based on their feedback, minor wording refinements were made to improve clarity without changing the content or structure of the questionnaire. Because the questionnaire consisted primarily of single-item factual, behavioral, service-exposure, and perception indicators rather than multi-item psychometric scales, internal consistency reliability was not applicable.

Sociodemographic variables included gender, age, ethnicity, education level, and monthly income. Age was grouped into three categories: <30 years, 30–44 years, and ≥45 years. Ethnicity was categorized as Hui, Achang, Bai, and other ethnic minority groups. Education level and monthly income were treated as ordered variables in the regression models. Education level was coded from 1 = below primary school to 5 = university or above, and monthly income was coded from 1 = <1,000 CNY to 4 = >5,000 CNY. The corresponding odds ratios therefore represented the change associated with moving to the next higher response category.

Health management variables included self-rated illness status, annual health check-up, free health check-up in the past year, delayed treatment, and understanding of chronic diseases. Health behavior items included electronic device use, weekly exercise, sleep duration, smoking, and drinking. These variables were used to describe the broader health profile of the participants. They were not entered into the primary hierarchical regression models because the inferential analysis was specifically structured around the study objectives: sociodemographic characteristics, health-service contact, chronic disease management perception, and digital health exposure. Variable inclusion followed this conceptual sequence rather than univariable statistical significance or automated variable-selection procedures.

Digital health exposure and perception variables included active attention to chronic disease-related websites, WeChat accounts, or apps; awareness of community-based internet-enabled (“Internet+”) chronic disease management services; and perceived importance of chronic disease management. In this study, community-based internet-enabled chronic disease management referred to services that used internet- or mobile-based tools to support health information, follow-up, or communication. These variables represented both individual exposure to digital health information and community-level exposure to digitally supported health services.

The primary outcome was EHR willingness. Participants were classified as willing or unwilling according to their response to the questionnaire item asking whether they were willing to establish an electronic health record. The binary outcome was coded as 1 = willing and 0 = unwilling for logistic regression analysis.

### 2.4 Statistical analysis

Descriptive statistics were used to summarize participant characteristics, health management gaps, digital health exposure, and EHR willingness. Categorical variables were reported as frequencies and percentages, and age was reported as mean ± standard deviation.

Binary logistic regression was used because EHR willingness was dichotomous and coded as 1 = willing and 0 = unwilling. A hierarchical modeling strategy was adopted to examine whether health-management and digital health-related factors explained additional variation in EHR willingness beyond sociodemographic characteristics. Variables were entered according to the conceptual sequence of the study rather than by automated selection.

Model 1 included sociodemographic characteristics: age group, gender, ethnicity, education level, and monthly income. Model 2 added health-management variables: self-rated illness status, annual health check-up, free health check-up in the past year, and understanding of chronic diseases. Model 3 further added digital health exposure and perception variables: attention to chronic disease-related digital tools, perceived importance of chronic disease management, and awareness of community-based internet-enabled chronic disease management services.

Because the exact size of the accessible target population could not be determined under the snowball sampling design, sample adequacy was evaluated according to the requirements of the principal inferential analysis rather than by using a finite-population formula. The final logistic regression model contained 15 regression coefficients. Using the smaller outcome category as a conservative basis, 259 participants provided approximately 17.3 outcome observations per coefficient. This exceeded the commonly used minimum criterion of 10 events per coefficient, indicating that the analytical sample was adequate for the planned logistic regression analysis (17).

Adjusted odds ratios, 95% confidence intervals, and *p* values were reported. Model performance was compared using Akaike information criterion, McFadden pseudo-R^2^, and the area under the receiver operating characteristic curve. A two-sided *p* value < 0.05 was considered statistically significant.

Complete data were available for all variables included in the regression analyzes; therefore, all three hierarchical models used the same analytical sample of 567 participants. Multicollinearity was assessed using variance inflation factors. Three sensitivity analyzes were conducted. First, education level and monthly income were treated as categorical rather than ordinal variables. Second, participants who selected “unclear” for the free health check-up item were excluded. Third, participants who selected “unclear” for the community internet-enabled chronic disease management service item were excluded. All other coding and model specifications remained unchanged.

### 2.5 Semi-structured resident interviews

Semi-structured interviews were conducted after completion of the quantitative survey and preliminary analysis to help explain selected statistical findings. All 21 interview participants had completed the survey and were included in the analytical sample of 567 participants. They were purposively recruited from respondents who completed the questionnaire through on-site, face-to-face administration at the three sampling locations. During on-site administration, research staff verbally invited respondents to participate in a follow-up interview; those who agreed provided a temporary contact number, which was stored separately from the analytical dataset and deleted after the interview phase. Selection aimed to include variation in EHR willingness, free health check-up experience, exposure to community internet-enabled chronic disease management services, and ethnic background.

The interview sample included 11 men (52.4%) and 10 women (47.6%), with ages ranging from 18 to 61 years, and represented different ethnic backgrounds within the survey population. Face-to-face interviews were conducted by the first author at the three sampling locations after participants provided separate consent for the interview and audio recording. Each interview lasted approximately 20–30 min and was audio-recorded with the participant’s permission.

Interviews were conducted primarily in Mandarin Chinese. Four older participants could understand Mandarin but had difficulty expressing themselves fluently. For these interviews, local bilingual assistants who spoke both Mandarin and the relevant local dialect assisted with interpretation during the interview. The recordings were initially transcribed using speech-to-text software. The corresponding author checked the automatically generated transcripts against the original recordings and corrected transcription errors before analysis. Illustrative quotations selected for presentation in the manuscript were translated from Chinese into English by the corresponding author and checked using back-translation to verify accuracy of meaning.

The interview guide was developed around the main quantitative findings. Interviews focused on residents’ experiences of routine health services, free health check-ups and follow-up, use of smartphones or health-related digital tools, understanding of chronic disease management, and expectations or concerns about electronic health records. Attention was paid to why some residents reported greater EHR willingness while others remained uncertain or unwilling.

Interview records were analyzed thematically. The first author conducted the initial coding and developed preliminary themes by comparing recurring meanings across transcripts. The corresponding author reviewed the transcripts, coding framework, theme definitions, representative quotations, and links between the qualitative themes and quantitative findings. Differences in interpretation were discussed until agreement was reached. Given the focused explanatory purpose of the interviews, sample adequacy was considered in terms of information sufficiency rather than formal theoretical saturation. Recruitment ended after 21 interviews because later interviews provided largely repetitive explanations and did not identify substantial new information relevant to the selected quantitative findings. Participants were anonymised using codes P01–P21, and representative quotations were selected for Table 1.

**Table 1:** Joint display integrating quantitative and qualitative findings on EHR willingness.

Quantitative result	Theme	Interview explanation	Illustrative quotation
Free health check-up: aOR = 2.14 (95% CI: 1.33–3.46)	Offline service access and continuity	Residents linked EHR willingness to prior contact with health checks and local services. However, some described health checks as distant, irregular, or lacking follow-up. Previous experiences of data collection without feedback reduced confidence in new digital health initiatives.	“We’ve had checkups and filled out forms before, but no one ever followed up with us about the results. We’d only find it useful if, after creating a health record, we could view the results and be notified about free follow-up exams.” (P02, female, 61, Hui, farmer, willing to establish an EHR)
Internet-enabled service awareness: aOR = 1.72 (95% CI: 1.16–2.55)	Practical barriers to using digital tools	Residents did not necessarily reject digital health services, but many reported difficulty using smartphones, apps, WeChat functions, or online platforms without help. This explained why digital health exposure depended on guidance and support rather than access alone.	“I only use my phone to answer calls and check messages; I do not know how to use apps. If I had to set up a profile on my phone, I definitely would not be able to do it without someone showing me how.” *(P09, male, 48, Achang, self-employed, no digital health exposure)*
Perceived importance: aOR = 2.41 (95% CI: 1.64–3.54); 45.7% unwilling	Perceived usefulness and feedback expectations	Residents reported greater EHR willingness when they believed an electronic record would help with chronic disease follow-up, check-up results, medication reminders, or future medical visits. Reluctance was mainly linked to unclear data use, lack of feedback, and uncertainty about practical benefits.	“If this form is just for filling out some information, I do not think it’s necessary. But if it can be used for future medical visits, checking health checkup results, or follow-up appointments for chronic conditions, then I’m willing to cooperate.” *(P13, female, 35, Bai, worker, conditional willingness)*

EHR = electronic health record. P = resident participant. Quotations are representative examples selected from resident interview transcripts.

### 2.6 Integration of quantitative and qualitative findings

Quantitative and qualitative findings were integrated at the interpretation stage using a joint display. The survey first described health management gaps, digital health exposure, and EHR willingness, and the regression analysis then identified associated factors. Interview themes were linked directly to the corresponding quantitative findings to clarify how service continuity, practical barriers to digital tool use, perceived usefulness, and expectations of feedback may have shaped the observed patterns. This integration supported a contextual interpretation of EHR willingness without implying causal relationships.

### 2.7 Ethical considerations

The study protocol (including two 17-year-old participants) was reviewed and approved by the Ethics Committee of Yunnan Minzu University [YXLL2026007]. Participants were informed of the study purpose, voluntary participation, anonymity, and confidentiality. Because the quantitative survey was administered electronically, informed consent for the survey was obtained through an electronic consent statement that participants confirmed by clicking to proceed before starting the questionnaire. Separate consent for the follow-up interview and audio recording was obtained from interview participants, who were told that they could decline to answer any question or withdraw at any time.

## 3 Results

### 3.1 Participant characteristics

A total of 567 valid responses were included in the analysis. The sample included slightly more men than women, and the mean age was 30.2 years. More than half of the participants were younger than 30 years, indicating a relatively young survey population. Hui participants accounted for the largest proportion of the sample, followed by Achang and Bai participants. Educational attainment was generally high, with most participants reporting a college diploma or university-level education. Monthly income was concentrated in the lower and middle categories, with 40.7% reporting a monthly income below 1,000 CNY and 30.7% reporting 1,000–3,000 CNY (Table 2).

**Table 2:** Participant characteristics (*N* = 567).

Variable	Category	*n*	(%)
Gender	Male	310	54.7
	Female	257	45.3
Age, years	Mean ± SD	30.2 ± 11.9	–
Age group	<30	317	55.9
Age group	30–44	153	27.0
Age group	≥45	97	17.1
Ethnicity	Hui	319	56.3
Ethnicity	Achang	115	20.3
Ethnicity	Bai	53	9.3
Ethnicity	Other	80	14.1
Education	Below primary school	18	3.2
Education	Primary school	30	5.3
Education	Middle school	50	8.8
Education	College diploma	308	54.3
Education	University or above	161	28.4
Monthly income, CNY	<1,000	231	40.7
Monthly income, CNY	1,000–3,000	174	30.7
Monthly income, CNY	3,000–5,000	85	15.0
Monthly income, CNY	>5,000	77	13.6

Percentages were calculated based on the analytical sample. Other ethnic groups included Yi, Jingpo, Miao, Zhuang, Dai, and other minority groups with small cell counts.

### 3.2 Health management gaps and digital health readiness

Table 3 presents indicators of health management gaps, digital health exposure, and EHR willingness. Several indicators suggested limitations in routine and preventive health management. More than half of the participants reported being occasionally or frequently ill, while 60.3% reported no annual health check-up. In addition, 72.3% reported either not receiving a free health check-up in the past year or being unclear about whether such services were available. A smaller but still notable proportion reported delaying treatment until symptoms became severe. These findings indicate that routine health monitoring and preventive service contact were uneven in this population.

**Table 3:** Health management gaps and digital health readiness among participants (*N* = 567).

Domain	Variable	Category	*n*	%
Health management gaps				
Health behavior	Electronic device use	>3 h/day	211	37.2
Health behavior	Weekly exercise	<2 times/week	106	18.7
Health behavior	Sleep duration	<7 h/day	226	39.9
Risk behavior	Any current smoking	Occasional or frequent smoking	282	49.7
Risk behavior	Any current drinking	Occasional or frequent drinking	364	64.2
Self-rated health	Illness status	Occasionally/frequently ill	289	51.0
Routine health management	Annual health check-up	No	342	60.3
Public health service	Free health check-up in past year	No/unclear	410	72.3
Healthcare-seeking	Delayed treatment	Endure symptoms until severe	136	24.0
Chronic disease knowledge	Understanding of chronic diseases	Do not understand	167	29.5
Digital health readiness				
Digital health exposure	Chronic disease website/WeChat/App	No active attention	217	38.3
Digital health exposure	Community internet-enabled chronic disease service	No/unclear	322	56.8
Digital health perception	Chronic disease management	Important	311	54.9
EHR willingness	EHR willingness	Willing	308	54.3
EHR willingness	EHR willingness	Unwilling	259	45.7

EHR = electronic health record. “Any current smoking” and “any current drinking” included both occasional and frequent smoking/drinking. “No/unclear” indicated either the absence of confirmed service exposure or uncertainty about service availability.

Indicators related to digital health exposure and EHR willingness also showed uneven readiness. Although slightly more than half of the participants regarded chronic disease management as important, 38.3% reported no active attention to chronic disease-related websites, WeChat accounts, or apps. More than half reported that community internet-enabled chronic disease management services were either unavailable or unclear to them. EHR willingness was moderate, with 54.3% willing and 45.7% unwilling. These results suggest that willingness cannot be assumed, even where digital health monitoring is considered relevant to chronic disease management.

### 3.3 Factors associated with EHR willingness

Table 4 shows the hierarchical logistic regression models predicting EHR willingness. Model performance improved after health-management and digital health-related variables were added. Compared with Model 1, Model 3 showed a lower AIC, a higher McFadden pseudo-R^2^, and a higher AUC, indicating improved model fit and discrimination.

**Table 4:** Hierarchical logistic regression models of factors associated with EHR willingness.

Variable	Model 1 aOR (95% CI)	Model 2 aOR (95% CI)	Model 3 aOR (95% CI)
Sociodemographic characteristics			
Age 30–44 years (ref. <30)	0.59 (0.36–0.97)^*^	0.66 (0.40–1.10)	0.63 (0.37–1.06)
Age ≥45 years (ref. <30)	0.58 (0.33–1.00)	0.64 (0.36–1.12)	0.67 (0.37–1.19)
Male (ref. female)	1.27 (0.87–1.84)	1.16 (0.79–1.69)	1.20 (0.81–1.78)
Achang ethnicity (ref. Hui)	4.33 (2.49–7.53)^***^	3.83 (2.16–6.80)^***^	3.85 (2.12–7.00)^***^
Bai ethnicity (ref. Hui)	3.79 (1.76–8.13)^***^	3.67 (1.68–8.00)^**^	3.40 (1.54–7.51)^**^
Other ethnicity (ref. Hui)	2.57 (1.41–4.69)^**^	2.22 (1.18–4.15)^*^	2.09 (1.09–4.00)^*^
Education level (per higher category)	1.13 (0.92–1.38)	1.12 (0.92–1.38)	1.10 (0.89–1.36)
Monthly income (per higher category)	0.85 (0.71–1.02)	0.88 (0.73–1.06)	0.89 (0.74–1.08)
Occasionally/frequently ill (ref. normal/healthy)	–	0.86 (0.59–1.25)	0.90 (0.61–1.32)
Health-management factors			
Annual health check-up (ref. no)	–	1.14 (0.77–1.70)	1.12 (0.75–1.69)
Free health check-up in past year (ref. no/unclear)	–	2.34 (1.47–3.71)^***^	2.14 (1.33–3.46)^**^
Understands chronic diseases (ref. no)	–	1.02 (0.67–1.55)	0.99 (0.64–1.52)
Digital health exposure and perception factors			
Attention to chronic disease digital tools (ref. none)	–	–	0.95 (0.63–1.42)
Chronic disease management perceived important (ref. optional/unimportant)	–	–	2.41 (1.64–3.54)^***^
Community internet-enabled chronic disease service (ref. no/unclear)	–	–	1.72 (1.16–2.55)^**^
AIC	696.4	688.2	666.7
McFadden pseudo-R^2^	0.132	0.153	0.188
AUC	0.735	0.752	0.78

The dependent variable was EHR willingness, coded as 1 = willing and 0 = unwilling. aOR = adjusted odds ratio; CI = confidence interval; AUC = area under the receiver operating characteristic curve. Model 1 included sociodemographic characteristics. Model 2 added health-management factors. Model 3 added digital health exposure and perception factors. ^*^p < 0.05, ^**^p < 0.01, ^***^p < 0.001. *N* = 567 for all three models.

In the final model, ethnicity remained associated with EHR willingness. Compared with Hui participants, Achang, Bai, and participants from other ethnic minority groups had higher odds of reporting willingness to establish an EHR. Age, gender, education level, and monthly income were not statistically significant in the final model.

Among the health-management variables, having received a free health check-up in the past year was independently associated with higher EHR willingness. By contrast, annual health check-up, self-rated illness status, and understanding of chronic diseases were not significant after adjustment. Among the digital health-related variables, perceived importance of chronic disease management and awareness of community internet-enabled chronic disease management services were associated with higher EHR willingness. Attention to chronic disease-related websites, WeChat accounts, or apps was not significant after adjustment. These findings indicate that EHR willingness was more closely associated with direct public health service contact, perceived relevance of chronic disease management, and community-level digital health service exposure than with individual online health information attention alone.

All variance inflation factors ranged from 1.03 to 1.63, indicating no evidence of problematic multicollinearity. Treating education level and monthly income as categorical variables did not materially alter the magnitude or statistical significance of the principal associations (likelihood-ratio *χ*^2^(5) = 10.50, *p* = 0.062). The findings were also robust to alternative handling of “unclear” responses. After excluding participants who selected “unclear” for free health check-ups, the association with EHR willingness remained significant (aOR = 2.43, 95% CI: 1.43–4.12, *p* = 0.001; *N* = 393). After excluding those who selected “unclear” for community internet-enabled chronic disease management services, the corresponding association also remained significant (aOR = 1.98, 95% CI: 1.24–3.16, *p* = 0.004; *N* = 414).

### 3.4 Qualitative explanations of key quantitative findings

Resident interviews were used to clarify why health service contact and digital health readiness were associated with EHR willingness. Three themes were identified from the interview data, as shown in Table 1.

First, residents connected EHR willingness with the continuity of offline health services. Some participants described health checks as distant, irregular, or lacking follow-up. Several residents had experienced previous health information collection or health checks but did not receive clear results or subsequent guidance. This helped explain why free health check-up experience was associated with higher EHR willingness: residents were more likely to value electronic records when they expected feedback, follow-up, or continued services rather than one-time data collection.

Second, practical barriers to using digital tools limited digital health readiness. Participants, particularly those less familiar with smartphones or apps, did not necessarily reject digital health services but reported difficulty using mobile applications, WeChat functions, or online platforms without assistance. This finding contextualized the survey result that many participants had limited digital health exposure and that community internet-enabled chronic disease management services were associated with EHR willingness.

Third, EHR willingness depended on perceived usefulness and expected feedback. Participants reported greater willingness when they believed an EHR could support chronic disease follow-up, access to check-up results, medication reminders, or future medical visits. Reluctance was mainly related to unclear data use, limited feedback, and uncertainty about whether electronic records would bring practical benefits. This explains why perceived importance of chronic disease management was associated with EHR willingness, while a substantial proportion of participants remained unwilling to establish an EHR.

## 4 Discussion

This study examined health management gaps, digital health exposure, and EHR willingness among ethnic minority residents with rural community backgrounds in Yunnan Province. Three findings are central to the study. First, routine and preventive health management remained uneven, as reflected in limited annual health check-ups, low exposure to free health check-up services, and incomplete awareness of community digital health services. Second, EHR willingness was moderate rather than universal, suggesting that digital health monitoring cannot be assumed to be acceptable simply because electronic records are technically available. Third, EHR willingness was associated with prior free health check-up experience, perceived importance of chronic disease management, and awareness of community internet-enabled chronic disease management services. The interview findings further showed that these associations were shaped by service continuity, practical ability to use digital tools, and expectations of feedback after health information collection.

Two features strengthen the study. First, it focuses on an underserved population for whom routine health-service access and digital support may be uneven. Second, the explanatory sequential design combines a relatively large survey sample with resident interviews, allowing statistical associations to be interpreted in relation to service experiences, practical barriers, and feedback expectations. These strengths should nevertheless be considered alongside the sampling and measurement limitations described below.

### 4.1 Routine service contact as the basis of digital health readiness

One important finding was that residents who had received a free health check-up in the past year had higher odds of reporting EHR willingness. This association may indicate that routine public health service contact makes digital health monitoring more concrete and service-related for residents. EHRs may be viewed more favorably when connected with health checks, follow-up, and local service experience rather than perceived as a separate data-collection exercise. This interpretation is consistent with the view that digital health interventions should be embedded in functioning health systems instead of introduced as stand-alone technical solutions (18, 19).

The interview data support this interpretation but do not establish causality. Some residents described previous experiences of health checks or form completion without later feedback, which appeared to weaken confidence in new health-information initiatives. These accounts suggest that visible offline services, understandable results, and follow-up arrangements may help residents recognize the practical role of EHRs in continuous health management.

### 4.2 Perceived usefulness and feedback expectations in EHR willingness

Perceived importance of chronic disease management was one of the strongest factors associated with EHR willingness. This finding is consistent with technology acceptance research, in which perceived usefulness and ease of use are repeatedly identified as important predictors of acceptance of health information systems (11, 20). However, the present study adds a contextual point: for rural ethnic minority residents, perceived usefulness was not only a technical judgment about whether an electronic record was convenient. It was closely tied to whether residents expected EHR to provide check-up results, follow-up reminders, chronic disease management support, or easier future medical visits.

This also helps explain why nearly half of the participants were unwilling to establish an EHR despite the potential benefits of digital health monitoring. The interviews suggested that reluctance was often conditional rather than absolute. Some residents were willing to establish an EHR if the record could be used for future medical visits or follow-up, but were less willing if it only meant filling in personal information. Previous studies have shown that trust and privacy concerns influence acceptance of health information technologies and willingness to allow health data to be used (10). In the present study, concerns about data use were accompanied by another practical concern: whether the information collected would be returned to residents through meaningful feedback.

### 4.3 Digital tools require practical support, not only availability

A notable result was that community internet-enabled chronic disease management services were associated with higher EHR willingness, whereas individual attention to chronic disease websites, WeChat accounts, or apps was not significant after adjustment. This contrast suggests that digital health engagement in rural ethnic minority communities may depend more on service-based digital exposure than on individual online information-seeking alone. Digital tools may become meaningful when they are linked to community services, local explanation, and practical support.

This interpretation is consistent with studies showing that digital health tools may help improve care access in underserved or rural settings, but their impact depends on implementation conditions, user support, and the ability of residents to engage with digital platforms (21). Studies on digital health literacy also show that the ability to seek, understand, and use digital health information is uneven, especially among groups with limited digital experience (22). The interviews in this study showed similar barriers. Some residents did not reject digital health, but reported that they did not know how to use apps, WeChat functions, or online health platforms without assistance. This suggests that digital health monitoring should be accompanied by low-threshold support from community workers, family members, or local health service providers.

### 4.4 Interpreting ethnic differences cautiously

Ethnicity remained associated with EHR willingness in the final model. This finding should be interpreted cautiously. It should not be read as evidence that ethnic identity itself determines acceptance of electronic health records. In this study, ethnic categories may also reflect differences in village location, health service contact, communication channels, mobility, community-level digital health exposure, and prior participation in public health programs. The interview findings also suggested that residents’ willingness was shaped by whether services were nearby, whether information was clearly explained, and whether health information collection was followed by feedback.

Therefore, ethnic differences in EHR willingness should be understood as possible indicators of uneven service contexts rather than as purely cultural differences. Future studies should collect more detailed information on distance to health services, village-level digital infrastructure, language and communication practices, and actual use of community health services. This would help distinguish ethnic-group differences from differences in service exposure and digital health support.

### 4.5 Public health implications

The findings suggest several implementation strategies that warrant evaluation rather than confirmed causal solutions. Community health workers could assist residents with EHR registration and explain privacy protection and data use before enrolment. Health check-up results could be returned in understandable formats, with follow-up reminders where appropriate. Village clinics or community service points could also serve as local digital health support hubs for residents who have difficulty using smartphones, apps, or WeChat-based services. These strategies may improve the practical relevance and accessibility of EHR implementation, but their effects on actual uptake should be tested in future intervention studies.

The central issue is therefore not only whether a digital health system can be technically developed, but whether it can be embedded in routine public health services in a form that residents can understand, use, and benefit from. In rural ethnic minority communities, EHR willingness may be shaped by service continuity, perceived usefulness, digital support, clear privacy communication, and visible feedback mechanisms.

### 4.6 Limitations

Several limitations should be noted. First, the cross-sectional design does not permit causal inference. Reverse causality is possible because residents who were already more interested in health management may have been more likely both to notice or recall service exposure and to report EHR willingness. Residual confounding related to recruitment site, local service availability, digital literacy, prior contact with community health workers, and other unmeasured contextual factors may also remain. Second, the non-probability snowball sample was relatively young and highly educated. QR-code-based recruitment may have underrepresented residents with limited smartphone access or lower digital literacy, while participant referral may have produced network homogeneity. Recruitment was initiated in Kunming, Dehong, and Honghe, and eastern Yunnan was not sampled. Moreover, because questionnaire links circulated through family and social networks, exact site-specific sample counts, referral waves, and the proportions recruited on site versus through referral were not available. These factors limit geographic coverage and prevent the findings from being interpreted as representative of all rural ethnic minority residents in Yunnan Province. Third, the questionnaire was researcher-developed and its items were analyzed individually rather than as validated multi-item scales, limiting interpretation of broader constructs such as digital health readiness. Fourth, combining “no” and “unclear” responses may have obscured meaningful differences between confirmed non-exposure and lack of awareness. Fifth, health behavior variables were used descriptively and were not included in the primary regression because the analysis focused on service contact, chronic disease management perceptions, and digital health exposure. Sixth, the qualitative phase was designed to explain selected quantitative findings rather than provide a comprehensive qualitative account; the 21 interviews, recruited only from on-site respondents, may not capture the full range of experiences across communities, and residents reached through onward snowball referral were not represented, which may have introduced some selection bias. Finally, the outcome item asked participants both whether they considered EHR establishment meaningful and whether they would be willing to cooperate, thereby combining perceived usefulness with behavioral willingness within a single question; this may limit the specificity of the outcome measure by not fully distinguishing these two dimensions. In addition, EHR willingness was self-reported and may differ from actual enrolment or sustained use when a digital health system is implemented. Although sensitivity analyzes excluding “unclear” responses produced consistent findings, the primary dichotomization may still have reduced measurement specificity because confirmed non-exposure and uncertainty may reflect different circumstances.

## 5 Conclusion

Among the surveyed ethnic minority residents with rural community backgrounds in Yunnan Province, health management needs coexisted with uneven readiness for digital health monitoring. EHR willingness was associated with prior free health check-up experience, perceived importance of chronic disease management, and awareness of community-based internet-enabled chronic disease management services. Resident interviews suggested that these associations may reflect service continuity, practical support for using digital tools, and expectations of visible feedback after health information collection. The findings support evaluating EHR implementation strategies that integrate routine public health services, community-level digital support, privacy communication, and feedback mechanisms rather than relying on a stand-alone technical system.

## Data Availability

The raw data supporting the conclusions of this article will be made available by the authors, without undue reservation.

## References

[1] Al Mahmud A Joachim S Jayaraman PP Learmonth C Tyagi S Forkan ARM . Digital health interventions to support chronic disease management: systematic scoping review. JMIR Mhealth Uhealth. (2026) 14:e63742. doi: 10.2196/63742 41533959 PMC12803440

[2] Haleem A Javaid M Singh RP Suman R. Telemedicine for healthcare: capabilities, features, barriers, and applications. Sens Int. (2021) 2:100117. doi: 10.1016/j.sintl.2021.100117 34806053 PMC8590973

[3] Rauner Y Stummer H. The socio-technical adoption and diffusion of digital health innovations: the development of the STAD-HC model based on telemedicine in Germany. Digit Bus. (2025) 5:100135. doi: 10.1016/j.digbus.2025.100135

[4] Radu I Scheermesser M Spiess MR Schulze C Händler-Schuster D Pehlke-Milde J. Digital health for migrants, ethnic and cultural minorities and the role of participatory development: a scoping review. Int J Environ Res Public Health. (2023) 20:6962. doi: 10.3390/ijerph20206962 37887700 PMC10606156

[5] Barrera M Jr Castro FG Strycker LA Toobert DJ. Cultural adaptations of behavioral health interventions: a progress report. J Consult Clin Psychol. (2013) 81:196–205. doi: 10.1037/a0027085 22289132 PMC3965302

[6] Wei S Long H Tian Y. Factors influencing healthcare-seeking behavior and its process analysis among ethnic minority residents: a qualitative study in rural China. Front Public Health. (2025) 13:1627392. doi: 10.3389/fpubh.2025.1627392 40620560 PMC12226278

[7] Yunnan Provincial Bureau of Statistics. (2021). Main data bulletin of the seventh national population census of Yunnan Province. Available online at: https://stats.yn.gov.cn/zwgk/zfxxgk/fdzdgknr/tjsj/tjgb/202105/t20210517_1204456.html (accessed August 19, 2026).

[8] Maita KC Maniaci MJ Haider CR Avila FR Torres-Guzman RA Borna S . The impact of digital health solutions on bridging the health care gap in rural areas: a scoping review. Perm J. (2024) 28:130–43. doi: 10.7812/tpp/23.134 39135461 PMC11404635

[9] Modi S Feldman SS. The value of electronic health records since the health information technology for economic and clinical health act: systematic review. JMIR Med Inform. (2022) 10:e37283. doi: 10.2196/37283 36166286 PMC9555331

[10] Belfrage S Helgesson G Lynøe N. Trust and digital privacy in healthcare: a cross-sectional descriptive study of trust and attitudes towards uses of electronic health data among the general public in Sweden. BMC Med Ethics. (2022) 23:19. doi: 10.1186/s12910-022-00758-z 35246118 PMC8896318

[11] Dhagarra D Goswami M Kumar G. Impact of trust and privacy concerns on technology acceptance in healthcare: an Indian perspective. Int J Med Inform. (2020) 141:104164. doi: 10.1016/j.ijmedinf.2020.104164 32593847 PMC7212948

[12] Ikram A Parveen S Vaportzis E. Perceptions of electronic health records by ethnically diverse groups in mental health: a systematic literature review. Glob Ment Health (Camb). (2025) 12:e110. doi: 10.1017/gmh.2025.10063 41080661 PMC12509162

[13] Macias-Konstantopoulos WL Collins KA Diaz R Duber HC Edwards CD Hsu AP . Race, healthcare, and health disparities: a critical review and recommendations for advancing health equity. West J Emerg Med. (2023) 24:906–18. doi: 10.5811/westjem.58408 37788031 PMC10527840

[14] Bober T Rollman BL Handler S Watson A Nelson LA Faieta J . Digital health readiness: making digital health care more inclusive. JMIR Mhealth Uhealth. (2024) 12:e58035. doi: 10.2196/58035 39383524 PMC11499716

[15] Matlin SA Hanefeld J Corte-Real A da Cunha PR de Gruchy T Manji KN . Digital solutions for migrant and refugee health: a framework for analysis and action. Lancet Reg Health Eur. (2025) 50:101190. doi: 10.1016/j.lanepe.2024.101190 39816782 PMC11732709

[16] United Nations General Assembly. Transforming Our World: The 2030 Agenda for Sustainable Development (A/RES/70/1). New York: United Nations (2015).

[17] Peduzzi P Concato J Kemper E Holford TR Feinstein AR. A simulation study of the number of events per variable in logistic regression analysis. J Clin Epidemiol. (1996) 49:1373–9. doi: 10.1016/s0895-4356(96)00236-3 8970487

[18] Enahoro Q Ogugua J Anyanwu E Akomolafe O Odilibe I Daraojimba A. The impact of electronic health records on healthcare delivery and patient outcomes: a review. World J Adv Res Rev. (2023) 21:451–60. doi: 10.30574/wjarr.2024.21.2.0478

[19] Tsai CH Eghdam A Davoody N Wright G Flowerday S Koch S. Effects of electronic health record implementation and barriers to adoption and use: a scoping review and qualitative analysis of the content. Life (Basel). (2020) 10:327. doi: 10.3390/life10120327 33291615 PMC7761950

[20] Rahimi B Nadri H Lotfnezhad Afshar H Timpka T. A systematic review of the technology acceptance model in health informatics. Appl Clin Inform. (2018) 9:604–34. doi: 10.1055/s-0038-1668091 30112741 PMC6094026

[21] MacAskill W Gill P Woloszczuk C Alam K Wallis K McGrail MR . A systematic review of the scope and impact of rural primary healthcare innovations using digital health technology. BMJ Open. (2026) 16:e097874. doi: 10.1136/bmjopen-2024-097874 PMC12863373 41611458

[22] Mukhtar T Babur MN Abbas R Irshad A Kiran Q. Digital health literacy: a systematic review of interventions and their influence on healthcare access and sustainable development Goal-3 (SDG-3). Pak J Med Sci. (2025) 41:910–8. doi: 10.12669/pjms.41.3.10639 40103887 PMC11911735

